# Comparison of a commercial water-gas shift catalyst and La modified Cu-based catalysts prepared by deposition-precipitation in methanol steam reforming

**DOI:** 10.55730/1300-0527.3415

**Published:** 2022-03-03

**Authors:** Orhan ÖZCAN, Ayşe Nilgün AKIN

**Affiliations:** Department of Chemical Engineering, Faculty of Engineering, Kocaeli University, Kocaeli, Turkey

**Keywords:** Hydrogen production, methanol steam reforming, sonochemical coprecipitation, lanthanum

## Abstract

Herein, a performance analysis of La-doped copper-based catalysts (CuO/ZrO_2_/La-Al_2_O_3_) in methanol steam reforming (MSR) was conducted and compared with a commercial low temperature water-gas shift catalyst (HiFUEL W220) to produce H_2_ with low CO selectivity. The physicochemical properties of as-obtained catalysts were characterized by N_2_ adsorption, XRD, and ICP-OES. Effect of calcination temperature (750 °C and 1000 °C) on the properties of mixed oxide support (La-Al_2_O_3_) were discussed based on catalytic activity. The optimum conditions of H_2_O/CH_3_OH ratio (1.0–3.0), space-time ratio (*W*_FA0_) (40–120 kg s mol^−1^), and reaction temperature (180–310 °C) were evaluated by a parametric study using the commercial catalyst (HF220). Additionally, thermodynamic equilibrium calculations of experimentally identified components by using Aspen HYSYS process simulation software were also performed to analyze MSR process. The results were indicated that the calcination temperature significantly affected the structural properties and the activity with respect to CO selectivity. An increasing trend in CO selectivity for catalysts with supports calcined at 750 °C and a decreasing trend for catalysts with supports calcined at 1000 °C were observed. Hence, CZ30LA_750_ and CZ30LA_1000_ catalysts were selected to attain low CO selectivity and comparable activity when compared to other catalysts and the simulated thermodynamic calculation results.

## 1. Introduction

Worldwide, renewable energy sources alternative to fossil fuels are being investigated by the scientific communities due to the growing concerns about environmental problems [1,2]. One of the promising and environmentally friendly options is the widespread implementation of hydrogen production technologies [3]. Hydrogen as an energy carrier has the advantage of reduced emissions of greenhouse gases in fuel cell applications, e.g., high-temperature polymer electrolyte membrane fuel cells (HT-PEMFC) [4]. However, hydrogen storage difficulties remain to be solved to power small-scale applications [5]. Therefore, distributed hydrogen production via reformers of various fuels is an attractive option to supply hydrogen for fuel cell systems like HT-PEMFC [6]. When compared to low-temperature PEMFC, high-temperature PEMFC can tolerate more fuel impurities and the waste heat can be recovered in a reformer-fuel cell integrated system [7]. Although most hydrogen is manufactured by methane today, other renewable liquid H_2_ carriers such as methanol have received much attention [8,9]. Compared to other fuels, methanol has moderate reforming temperatures (200–300 °C), high H/C ratio, and easy storage and transport advantages [10]. Furthermore, renewable methanol (biomethanol) can be produced from biomass resources through various processes [11]. Among other reforming techniques (partial oxidation and autothermal reforming of methanol), endothermic reaction of methanol steam reforming (MSR) gives the highest H_2_ yield (3 mol H_2_ per mol of CH_3_OH) as presented in Equation (1) [12]. In MSR process, some side reactions can also occur such as methanol decomposition (MD) in Equation (2), water-gas shift (WGS) reaction in Equation (3), and reverse water-gas shift (RWGS) reaction in Equation (4) [13].


(1) $${\text{CH}}_{3}\text{OH}+{\text{H}}_{2}\text{O}⇄{\text{CO}}_{2}+3{\text{H}}_{2}\;\;\;\Delta {\text{H}}_{298\;\text{K}}=49\;\text{kJ}/\text{mol}$$

(2) $${\text{CH}}_{3}\text{OH}⇄\text{CO}+2{\text{H}}_{2}\;\;\;\Delta {\text{H}}_{298\;\text{K}}=90\;\text{kJ}/\text{mol}$$

(3) $$\text{CO}+{\text{H}}_{2}\text{O}⇄{\text{CO}}_{2}+{\text{H}}_{2}\;\;\;\Delta {\text{H}}_{298\;\text{K}}=-41\;\text{kJ}/\text{mol}$$

(4) $${\text{CO}}_{2}+{\text{H}}_{2}⇄\text{CO}+{\text{H}}_{2}\text{O }\;\;\Delta {\text{H}}_{298\;\text{K}}=41\;\text{kJ}/\text{mol}$$

Choice of catalysts affects the activity and selectivity of MSR system [14]. Extensive use of inexpensive Cu-based catalysts in MSR was reported in the literature with high activity but low thermal and long-term stability [15]. To eliminate the drawback of copper-based catalysts, highly stable group 8–10 metals, mostly palladium, were also studied widely and reported to produce synthesis gas mostly composed of H_2_ and CO [16]. Moreover, effects of promoters in addition to different catalyst preparation techniques, mainly coprecipitation, were investigated to enhance the catalytic properties of copper-based catalysts [17]. In recent years, sonochemical coprecipitation has been adopted to enhance physicochemical properties of the final sample by a mechanical effect leading to better dispersion of species [18,19]. Generally, commercial Cu-based catalysts for MSR process consist of ZnO as a promoter [20]. However, effects of different promoters such as ZrO_2_, SiO_2_, Y_2_O_3_, CeO_2_, etc., were explored in MSR by several researchers to obtain more active catalysts [21–23]. It was reported that the addition of zirconia into the copper-based catalysts is beneficial by increasing the surface area and decreasing the possibility of Cu sintering [24]. Ternary Cu/ZnO/X models in comparison with binary Cu/ZnO systems were employed in the study of Alejo et al. by preparing a series of Cu_40_Zn_60_ and Cu_40_Zn_55_Al_5_ catalysts [25]. They concluded that the highly stable catalysts were evaluated in the presence of alumina even after 110 h operation time where Cu_40_Zn_60_ was deactivated after 20 h. Thus, modification of second oxide phase, X, could exhibit a potential to increase MSR activity [26]. Lanthanum is an attractive dopant to contribute more stabilized oxide lattice with its strong binding to oxygen [27,28]. In a reported work of Papavasiliou et al., various metal oxides of La, Zr, Mg, Gd, Y, and Ca were studied in a Cu/CeO_2_ system [29]. The results were indicated that CO selectivity was lowered for a part of promoters including lanthanum. Additionally, Lu et al. has studied the influences of La on a Ni-based catalyst. As far as CO selectivity was concerned, a decrease was reported with the incorporation of lanthanum by helping separate the NiO particles with high dispersion [30].

In the present study, a commercial Cu-based low temperature water-gas shift (WGS) catalyst (HiFUEL W220) was used to find optimum operational parameters of MSR system in the range of practical interest. The selection of the catalyst HiFUEL W220 (hereafter mentioned as HF220) was due to its composition, which also acts as a successful MSR catalyst. Preliminary performance tests of commercial catalyst were conducted by considering the effects of H_2_O/CH_3_OH ratio, W/F_A0_, and temperature on product composition. In addition, Cu-ZrO_2_/La-Al_2_O_3_ ternary catalysts with increasing lanthanum oxide weight percentages were prepared by ultrasound-assisted coprecipitation method. All the activity tests were performed at optimal conditions of MSR. Also, thermodynamic equilibria were calculated by using Aspen HYSYS software [31]. All the results were discussed based on equilibrium calculations with regard to CO formation to assess the activities of in-house catalysts.

## 2. Materials and methods

### 2.1. Support and catalyst preparation

Preparation of support materials (La-Al_2_O_3_) with increasing La_2_O_3_ content (0/10/20/30/40/50 wt.%) was performed by an ultrasound-introduced coprecipitation method. Also, ultrasound-assisted deposition-precipitation of copper and zirconium onto the supports was done in one step. Furthermore, a commercial low temperature water-gas shift catalyst, CuO/ZnO/Al_2_O_3_ (HiFUEL W220), was purchased from Alfa Aesar to compare with lanthanum modified in-house catalysts. The catalyst precursors (La(NO_3_)_3_.6H_2_O (Sigma Aldrich), Al(NO_3_)_3_.9H_2_O (Merck), Cu (NO_3_)_2_.3H_2_O (Merck), ZrO(NO_3_)_2_.xH_2_O (Sigma Aldrich)) were purchased and used as received. Briefly outlining, in the first step of support preparation, a 200 mL aqueous solution of the precipitating agent Na_2_CO_3_ (Merck), was heated to 70 °C under continuous stirring on a temperature-controlled stir plate. Another mixture containing La(NO_3_)_3_ and Al(NO_3_)_3_ was stirred in 200 mL of deionized (DI) water and added dropwise into the previously prepared solution under ultrasound irradiation (90W) using Bandelin Sonopuls HD3200. The pH of the final mixture was set in the range of 8–9 at 70 °C with a 2M NaOH (Merck). After aging process at 70 °C for 20 h, filtering and washing with deionized water were done to obtain the precipitates. Following the drying at 110 °C for 18 h, calcination was performed to obtain the mixed oxide supports at two different temperatures of 750 °C and 1000 °C for 4 h (heating rate 5 °C/min). All the support materials prepared by sonochemical coprecipitation were presented in Table 1.

**Figure 1:**
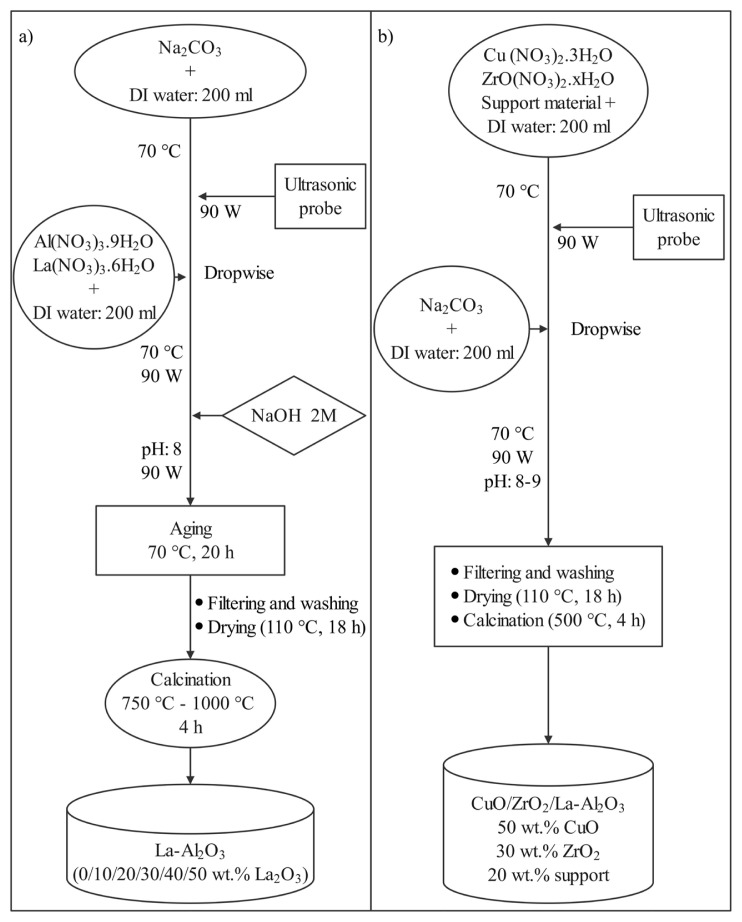
Preparation procedures for a) support and b) catalyst.

Similarly, two different solutions were mixed separately to prepare the Cu-based MSR catalysts. Firstly, powders of desired amounts of La modified support were mixed with aqueous solutions of Cu (NO_3_)_2_ and ZrO(NO_3_)_2_ (solution 1) and heated to 70 °C. Solution 2 was prepared by dissolving Na_2_CO_3_ in 200 mL of DI water and added into solution 1 drop by drop under ultrasound (90 W). Filtering, washing, and drying were accomplished under the same conditions of the support preparation process. Finally, calcination was conducted at 500 °C for 4 h with a ramp rate of 5 °C/min in a muffle furnace. All the in-house catalysts prepared by sonochemical deposition-precipitation were listed in Table 2. Also, the aforementioned preparation procedures of the supports and catalysts were summarized in the panels a and b of Figure 1, respectively.

### 2.2. Characterization

The characterization of the selected calcined catalysts was achieved by Brunauer-Emmet-Teller (BET), X-ray diffraction (XRD), and inductively coupled plasma optical emission spectrometry (ICP-OES). Measurements of BET surface area and adsorption-desorption isotherms at 77 K were performed on a Micromeritics ASAP 2020 instrument. The samples were out-gassed under vacuum at 473 K for 2 h before adsorption analysis. The XRD spectra were collected on a Rigaku MiniFlex II diffractometer operating at 30 kV and 15 mA with Cu Kα radiation source (λ = 0.154 nm). The XRD patterns were collected at 2θ angles (10–80°) with a scanning rate of 2°/min at ambient conditions. The diffraction patterns were analyzed using PDXL software (Rigaku Inc.) and the Crystallography Open Database [32]. The metal content of selected samples was determined by ICP-OES on a Perkin Elmer Optima 4300DV instrument.

### 2.3. Experimental set-up

**Figure 2:**
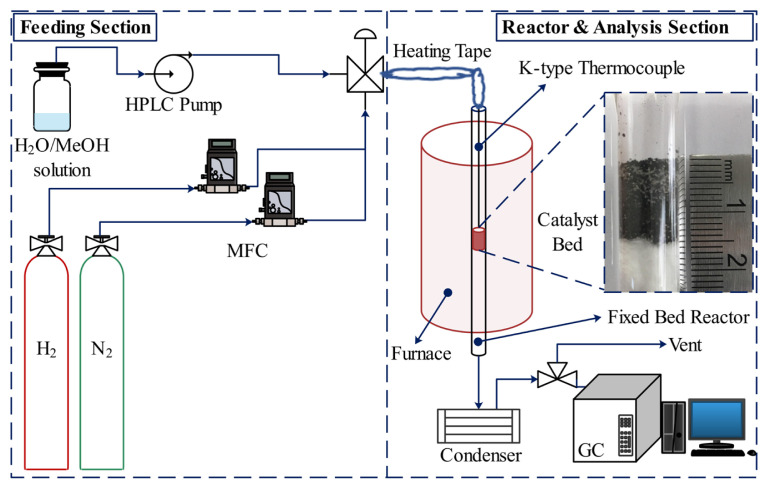
Catalytic activity test system.

**Figure 3:**
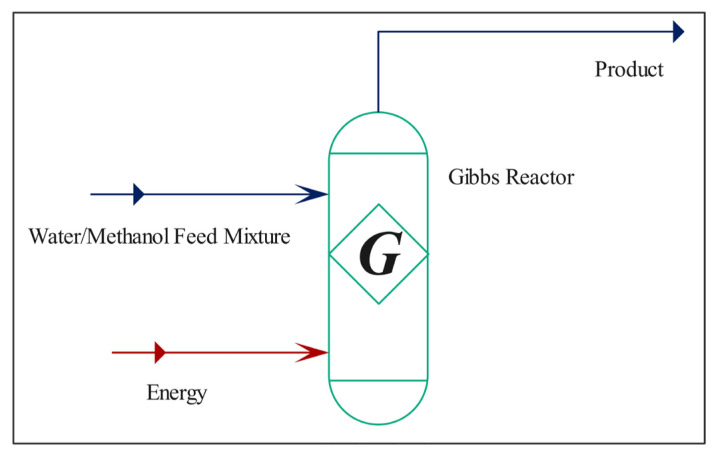
HYSYS simulation flow diagram for thermodynamic analysis.

The MSR catalytic performance tests of HF220 and all La-modified catalysts were carried out in a fixed bed tubular reactor (10 mm i.d., 50 cm length) in a built set-up as schematically given in Figure 2. Ideal plug-flow pattern was ensured with a reactor diameter to particle diameter ratio greater than 30 (*d_tube_*/*d_particle_* ≥ 30) and reactor tube length to particle diameter ratio greater than 50 (*L_tube_*/*d_particle_* ≥ 50). In all runs, 450 mg of fresh catalyst particles (45–60 mesh) diluted with 900 mg of inert quartz powder (80–100 mesh) were loaded on quartz wool inside the reactor. A type K thermocouple was placed in the center of the bed to monitor the reaction temperature. To achieve metallic Cu particles, all catalysts were reduced in situ with 80 vol.% H_2_/N_2_ flow (50 mL/min) at 330 °C for 60 min and cooled to the reaction temperature of 246 °C under pure N_2_ flow (10 mL/min). In the feeding section, the reactant liquids (a mixture of H_2_O and CH_3_OH) and the gases (H_2_ and N_2_) were dosed by an HPLC pump and calibrated thermal mass flow controllers (Teledyne Hastings HFC202), respectively. The liquid mixture was vaporized and introduced into the reactor by flowing N_2_ through a heating belt operating at 170 °C with a programmable controller. The effluent gases were fed through an ice-cooled condenser to ensure a water-free reaction mixture prior to analysis of the products, H_2_, CO_2_, CO, and N_2_ by online gas chromatography (GC) (Agilent 7890B) equipped with TCD and FID detectors. On the basis of the experimental results, methanol conversion along with the selectivities of H_2_, CO, and CO_2_ were defined as below


(5) $$X_{{\text{CH}}_{3}\text{OH}}(\%)=\frac{F_{{\text{CO}}_{2}}^{out}+F_{\text{CO}}^{out}}{F_{{\text{CH}}_{3}\text{OH}}^{in}}\times 100$$

(6) $$S_{{\text{H}}_{2}}(\%)=\frac{F_{{\text{H}}_{2}}^{out}}{F_{{\text{H}}_{2}}^{out}+F_{{\text{CO}}_{2}}^{out}+F_{\text{CO}}^{out}}\times 100$$

(7) $$Y_{{\text{H}}_{2}}(\%)=X_{{\text{CH}}_{3}\text{OH}}\times S_{{\text{H}}_{2}}(\%)$$

(8) $$S_{{\text{CO}}_{\text{X}}}(\%)=\frac{F_{{\text{CO}}_{\text{X}}}^{out}}{\Sigma \;F_{{\text{CO}}_{\text{X}}}^{out}}\times 100$$

(9) $$Y_{\text{CO}}(\%)=\frac{F_{\text{CO}}^{out}}{F_{{\text{CH}}_{3}\text{OH}}^{in}}\times 100,$$

where *F* is the flow rates of components with its respective subscript, Y_H2_ is the hydrogen yield and S_CO_ is the CO yield. Additionally, S_H2_ and S_COX_ are the selectivities towards H_2_ and CO_x_ compounds, respectively. In the first part of this study, a parametric optimization study of process variables i.e. H_2_O/CH_3_OH ratio (1.0–3.0), space-time ratio (*W*_FA0_) (40–120 kg s mol^−1^), and reaction temperature (180–310 °C) was conducted over the commercial catalyst, HF220. Also, performance analysis of La-doped Cu-based catalysts was evaluated at the optimized conditions based on activity results. Furthermore, a thermodynamic analysis approach described in detail elsewhere in our previous study was used in all runs to understand the effect of reaction parameters on MSR reactions [33,34]. Herein, Aspen HYSY simulation software was used to calculate equilibrium compositions of CH_3_OH, H_2_O, CO_2_, CO, and H_2_ (experimentally identified) understudied conditions. The flow diagram of the simulation to conduct thermodynamic calculations were indicated in Figure 3.

## 3. Results and discussion

### 3.1. Physical characterization

**Figure 4:**
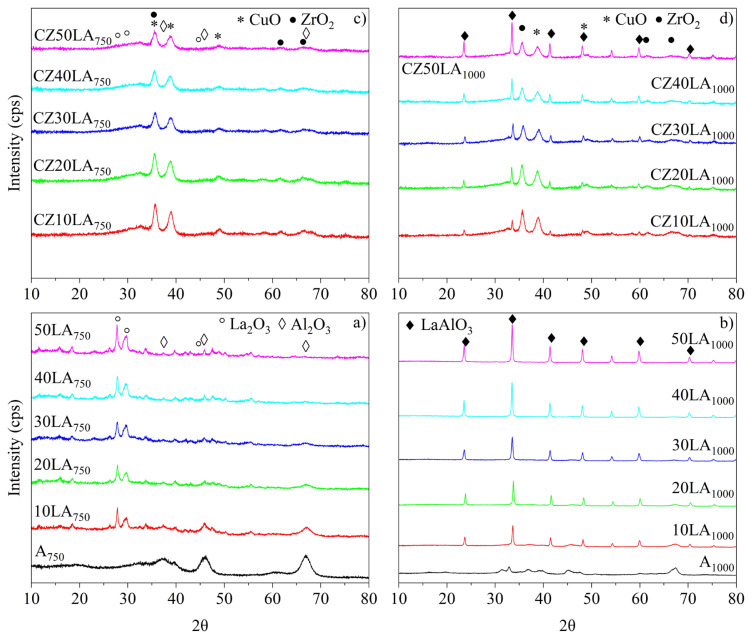
XRD patterns of a) calcined supports at 750 °C, b) calcined supports at 1000 °C, c) metal-loaded catalysts on supports (750 °C), d) metal-loaded catalysts on supports (1000 °C).

X-ray diffraction (XRD) patterns of supports calcined at 750 °C and metal-loaded catalysts were shown in panels a and c of Figure 4. Additionally, XRD results of supports calcined at 1000 °C and catalysts with copper and zirconium were given in panels b and d of Figure 4. As seen from Figure 4a, the characteristic peaks of Al_2_O_3_ at 37.40, 45.93, and 66.84 2θ in agreement with Crystallography Open Database (COD) card number 1200015 were identified to be decreasing with increasing La content. The gradually disappearing intensities of aluminum oxide peaks were attributed to homogeneously dispersed particles in the structure of La_2_O_3_ at 26.23, 29.66, and 45.79 (COD number 1010278). However, when the calcination temperature of La-doped supports was raised from 750 °C to 1000 °C, a phase transformation of the identified La_2_O_3_ peaks to LaAlO_3_ perovskite-like bulk structure (card number 5910090) was seen in Figure 4b. After copper and zirconium were loaded on the supports via ultrasound-assisted deposition-precipitation, CuO (card number 1011194) and ZrO_2_ (card number 1538970) were successfully observed in all catalysts. The diffraction peaks for CuO (35.65, 38.86, 48.79) and ZrO_2_ (35.65, 61.65, 66.67) were depicted in panels c and d of Figure 4.

**Figure 5:**
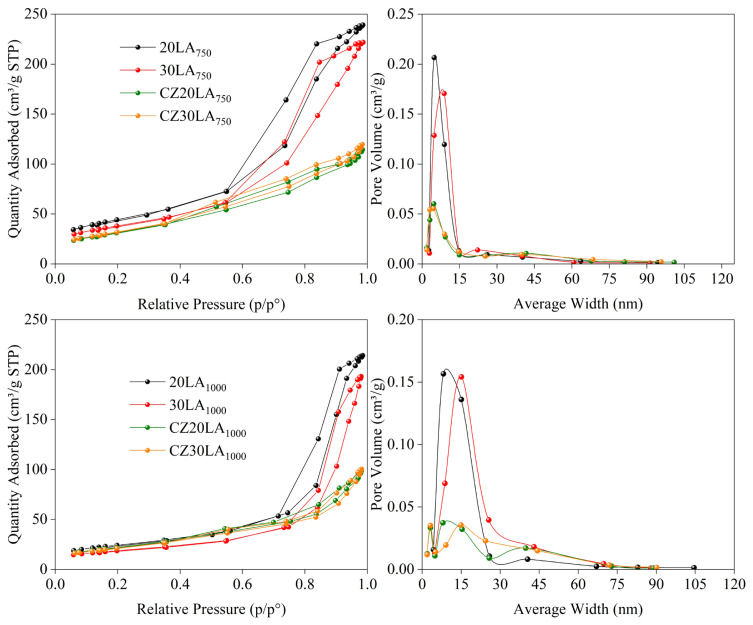
Adsorption-desorption isotherms with pore volume distributions of selected samples.

When the commercial HiFUEL W220 (HF220) was considered, X-ray diffraction peaks of CuO were observed prior to the reduction process at 330 °C where metallic Cu was obtained. Surface area, adsorption-desorption isotherms, and composition of the chosen catalysts were determined via BET and ICP-OES. N_2_-physisorption analysis was performed on the selected supports and catalysts due to their relatively low CO selectivities (see section ‘Catalytic reactivity’) and summarized in Table 3. As presented in Table 3, surface areas of catalysts were found to be decreasing owing to possible blockage of pore volumes with the addition of Cu and Zr. When the support samples at 750 °C and 1000 °C were compared, a drastic decrease in surface area was observed at 1000 °C and attributed to the formation of bulk LaAlO_3_ perovskite-like structure. The adsorption-desorption isotherms with pore volume distributions of the selected samples were illustrated in Figure 5. In Figure 5, all the supports (20LA_750_, 30LA_750_, 20LA_1000_, 30LA_1000_) were exhibited similar type IV isotherms with H2 hysteresis loop based on the IUPAC classification indicating the mesoporous structure was achieved successfully after calcination. In addition, all support materials were showed similar results in terms of average pore width and pore volume distribution. However, by the inclusion of copper and zirconium onto the supports, a noticeable reduction in pore volume distribution of the catalysts (CZ20LA_750_, CZ30LA_750_, CZ20LA_1000_, CZ30LA_1000_) was seen due to the metals filling the pores. This result was consequently generated a transition of hysteresis loops from H2 (observed on supports) to H3 type (observed on catalysts) implying the layered aggregation of loaded particles. It should be noted that the weight percentages of CuO (50 wt.%) and ZrO_2_ (30 wt.%) were considerably high when compared to mixed oxide support (20 wt.%) which may favor the agglomeration on the catalyst. The ICP-OES results of randomly selected support (10LA_1000_) and catalysts (CZ50LA_750_ and CZ50LA_1000_) along with the composition of commercial catalyst (HF220) (provided by the manufacturer) were given in Table 4. A good agreement was attained with the targeted experimental compositions when compared to weight percentages determined by ICP-OES. It is a well-established fact that Cu-species play an important role for being the active sites in MSR process [4]. Therefore, in all prepared catalysts, a very close CuO loading was selected when compared to HF220 in order to minimize the variability of activity results.

### 3.2. Catalytic reactivity

**Figure 6:**
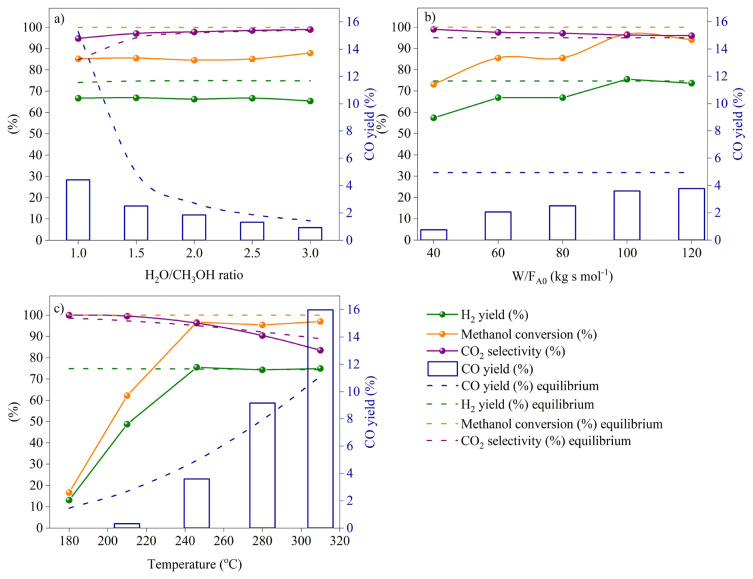
Effect of process variables on methanol conversion, H_2_ and CO yields, and CO_2_ selectivity. Experimental conditions: a) T = 246 °C, W/F_A0_ = 80 kg s mol^−1^, Time = 90 min. b) T = 246 °C, H_2_O/CH_3_OH = 1.5, Time = 90 min. c) W/F_A0_ = 100 kg s mol^−1^, H_2_O/CH_3_OH = 1.5, Time = 90 min.

Optimum methanol steam reforming (MSR) process parameters (H_2_O/CH_3_OH, space-time ratio (*W*_FA0_), reaction temperature) in the range of interest were determined on the commercial catalyst, HF220, by changing one parameter at a time in each set of experimental runs. The evaluation of performance analysis of all prepared La-doped Cu-based catalysts was done at the optimal operative conditions. The combined effect of process variables on methanol conversion, H_2_ and CO yields, and CO_2_ selectivity with equilibria were depicted in panels a–c of Figure 6. In Figure 6a, the product distribution of the MSR system was given as a function of the H_2_O/CH_3_OH ratio at 246 °C with a space-time ratio of 80 kg s mol^−1^. It was recognized that CO yield was decreased with increasing water content where steam reforming [Equation (1)] outweighs other reactions in the system such as decomposition [Equation (2)]. However, no considerable effects were identified for CH_3_OH conversion, H_2_ yield, and CO_2_ selectivity with excessive inclusion of water in the reactant mixture. Hence, considering the energy requirement to vaporize the liquid feed, increasing H_2_O/CH_3_OH molar ratio above 2 (stoichiometric ratio is 1) seems not feasible when a fuel reformer and a fuel cell integrated system is considered [35]. Thus, an optimum value of 1.5 for steam-to-methanol ratio was selected for further experimental runs. The effect of space-time ratio (Figure 6b) on product distribution using the commercial HF220 catalyst was investigated at 246 °C. It is worth mentioning that the reason why reaction temperature of 246 °C was chosen for the analysis of steam-to-methanol and space-time ratios is due to our recent study on thermodynamic study of MSR [33]. In Figure 6b, space-time ratio was increased by decreasing the total flow of water-methanol feed mixture. It was illustrated that increasing the space-time ratio was positively affected the conversion and yields where a plateau was seen after 100 kg s mol^−1^. This result can be explained by the fact that at high space-time values the reactants contact more with Cu-particles. Also, at low space-time values, the reactants with high flow rates may have encountered some diffusion limitations of interparticle or intraparticle. A slight increase in CO yield was also seen in Figure 6b due to endothermic reverse water-gas shift reaction (RWGS) [Equation (4)] that become more favored at the reaction temperature of 246 °C. Therefore, prior to the temperature effect study, the space-time ratio was selected 100 kg s mol^−1^ where the conversion reaches its highest values. The effect of reaction temperature on MSR process (Figure 6c) was examined in the range of 180–310 °C at abovementioned optimum conditions. Climbing methanol conversion and H_2_ yield were depicted up to the thermodynamically optimum temperature of 246 °C and kept constant above. Nevertheless, with increasing reaction temperature above 246 °C, an undesirable increase in CO yield was experienced that is detrimental for anode catalyst of a fuel cell. When a successful integration of a methanol reformer with a high temperature polymer electrolyte fuel cell (HT-PEMFC) is regarded, design and preparation of successful MSR catalysts are required especially at low temperatures.

**Figure 7:**
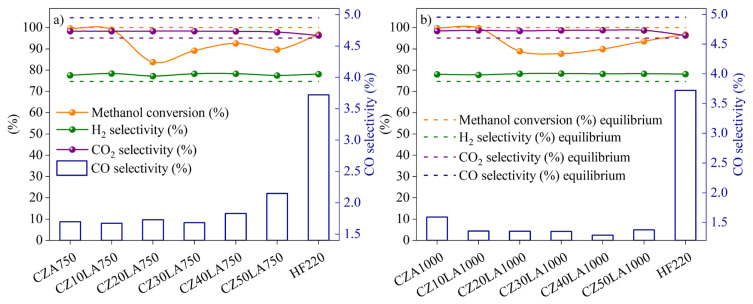
Reactivity results of HF220 and the catalysts with La-doped supports calcined at a) 750 °C, b) 1000 °C. Experimental conditions: T = 246 °C, W/F_A0_ = 100 kg s mol^−1^, H_2_O/CH_3_OH = 1.5, Time = 90 min.

**Figure 8:**
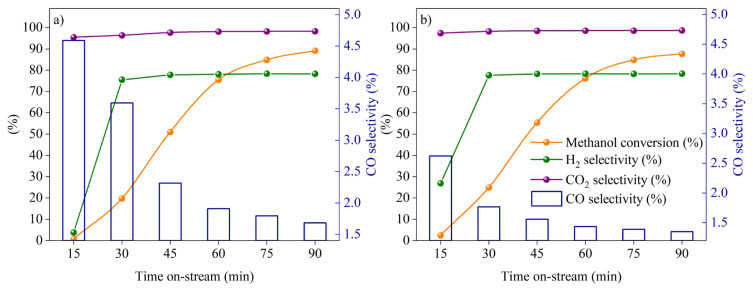
Time on-stream behavior of a) CZ30LA_750_ and b) CZ30LA_1000_ catalysts at 246 °C, W/F_A0_ = 100 kg s mol^−1^, H_2_O/CH_3_OH = 1.5.

The equilibrium conditions and the reactivity results of HF220 and the catalysts with La-doped supports calcined at 750 °C and 1000 °C were given in Figures 7a and 7b, respectively. All the samples were generally exhibited high conversion of methanol and selectivities of H_2_ and CO_2_. However, the CO selectivity was experienced an increasing trend in Figure 7a and a decreasing trend in Figure 7b for the prepared catalysts. As specified earlier in subsection ‘Physical characterization’, support materials with 1000 °C calcination temperature are more in bulk structure (LaAlO_3_) when compared to samples calcined at 750 °C. Given the aforementioned conclusion, this bulk structure could affect the metal-support interaction and prevent the formation of copper aluminates. Therefore, La as a promoter may contribute to increasing active site distribution and decreasing the CO selectivity which is in good agreement with open literature [36]. However, in Figure 7 a CO selectivity was increased after CZ30LA_750_ with the addition of more lanthanum to the support. Therefore, CZ30LA_750_ and CZ30LA_1000_ catalysts were found to be promising in terms of lower CO selectivities than the commercial catalyst (HF220) with their comparable activities. The reactivity results of selected catalysts in comparison with HF220 and equilibrium conditions were summarized in Table 5. As seen, the commercial catalyst was aligned well with the thermodynamic data indicating that the rates are sufficiently high to achieve equilibrium conversions. Nevertheless, the variability in the activities of in-house catalysts of CZ30LA_750_ and CZ30LA_1000_ may be due to the possibility of diffusion limitations. Furthermore, time on-stream data of the prepared catalysts of CZ30LA_750_ and CZ30LA_1000_ for 90 min reaction time were depicted in Figures 8a and 8b, respectively.

## 4. Conclusion

In this study, methanol steam reforming (MSR) activities of in-house La-doped Cu-based catalysts (CuO/ZrO_2_/La-Al_2_O_3_) with increasing lanthanum content and commercial CuO/ZnO/Al_2_O_3_ (HiFUEL W220) catalyst were investigated. Also, the effect of La addition to the structure of aluminum oxide support with two different calcination temperatures of 750 °C and 1000 °C was explored based on characterization and reactivity results. Furthermore, the performances of catalysts were compared with the calculated equilibrium conditions of MSR by Aspen HYSYS software. The main conclusions obtained in this work are summarized below:

- In XRD patterns of supports calcined at 1000 °C, the bulk structure of LaAlO_3_ phases was attained. In addition, La_2_O_3_ and Al_2_O_3_ phases were observed in XRD patterns of supports calcined at 750 °C with higher surface area when compared to supports calcined at 1000 °C. Also, CuO and ZrO_2_ phases were identified successfully for all catalysts.
- The ICP-OES analysis results of randomly selected support (10LA_1000_) and catalysts (CZ50LA_750_ and CZ50LA_1000_) were shown good agreement when compared with targeted weight percentages.
- Favorable conditions of the variables to give high methanol conversion and low energy consumption were investigated on the commercial catalyst (HF220) and found to be 1.5 (H_2_O/CH_3_OH ratio), 100 kg s mol^−1^ (space-time ratio), and 246 °C (reaction temperature).
- For all samples, CO selectivities were lower than thermodynamic analysis results. The decreasing trend in CO selectivity on the catalysts with supports calcined at 1000 °C was attributed to the generation of a possible beneficial synergy effect between Cu and the mixed oxide support LaAlO_3_. The results were demonstrated that CZ30LA_750_ and CZ30LA_1000_ catalysts are promising to minimize CO formation and reach high activity in MSR process.

## Figures and Tables

**Table 1:** Lanthanum doped mixed-oxide supports.

Supports	Name	Calcination temperature (°C)
Al_2_O_3_	A_750_	750
Al_2_O_3_	A_1000_	1000
10wt.% La_2_O_3_-Al_2_O_3_	10LA_750_	750
10wt.% La_2_O_3_-Al_2_O_3_	10LA_1000_	1000
20wt.% La_2_O_3_-Al_2_O_3_	20LA_750_	750
20wt.% La_2_O_3_-Al_2_O_3_	20LA_1000_	1000
30wt.% La_2_O_3_-Al_2_O_3_	30LA_750_	750
30wt.% La_2_O_3_-Al_2_O_3_	30LA_1000_	1000
40wt.% La_2_O_3_-Al_2_O_3_	40LA_750_	750
40wt.% La_2_O_3_-Al_2_O_3_	40LA_1000_	1000
50wt.% La_2_O_3_-Al_2_O_3_	50LA_750_	750
50wt.% La_2_O_3_-Al_2_O_3_	50LA_1000_	1000

**Table 2:** Cu-based catalysts prepared by sonochemical deposition-precipitation.

Catalysts	Name	Calcination temperature (°C)
50wt.% CuO/30wt.% ZrO_2_/20wt.% (A_750_)	CZA_750_	500
50wt.% CuO/30wt.% ZrO_2_/20wt.% (A_1000_)	CZA_1000_	
50wt.% CuO/30wt.% ZrO_2_/20wt.% (10LA_750_)	CZ10LA_750_	
50wt.% CuO/30wt.% ZrO_2_/20wt.% (10LA_1000_)	CZ10LA_1000_	
50wt.% CuO/30wt.% ZrO_2_/20wt.% (20LA_750_)	CZ20LA_750_	
50wt.% CuO/30wt.% ZrO_2_/20wt.% (20LA_1000_)	CZ20LA_1000_	
50wt.% CuO/30wt.% ZrO_2_/20wt.% (30LA_750_)	CZ30LA_750_	
50wt.% CuO/30wt.% ZrO_2_/20wt.% (30LA_1000_)	CZ30LA_1000_	
50wt.% CuO/30wt.% ZrO_2_/20wt.% (40LA_750_)	CZ40LA_750_	
50wt.% CuO/30wt.% ZrO_2_/20wt.% (40LA_1000_)	CZ40LA_1000_	
50wt.% CuO/30wt.% ZrO_2_/20wt.% (50LA_750_)	CZ50LA_750_	
50wt.% CuO/30wt.% ZrO_2_/20wt.% (50LA_1000_)	CZ50LA_1000_	

**Table 3:** N_2_-physisorption results of selected supports and catalysts.

Supports and catalysts	BET surface area (m^2^/g)	BJH desorption cumulative volume of pores (cm^3^/g)	BJH desorption average pore width (nm)
20LA_750_	159.8	0.37	5.9
CZ20LA_750_	113.6	0.18	4.5
30LA_750_	137.1	0.35	6.8
CZ30LA_750_	116.4	0.19	4.5
20LA_1000_	87.8	0.33	10.2
CZ20LA_1000_	79.9	0.16	5.8
30LA_1000_	77.4	0.30	13.2
CZ30LA_1000_	67.6	0.16	6.2

**Table 4:** ICP-OES results of randomly selected samples with the composition of commercial catalyst.

Sample	Chemical composition (wt.%) (ICP-analysis)	Chemical composition (wt.%) (Target)
10LA_1000_	La_2_O_3_ = 8.9	La_2_O_3_ = 10
	Al_2_O_3_ = 79.4	Al_2_O_3_ = 90
CZ50LA_750_	CuO = 50.0	CuO = 50
	ZrO_2_ = 28.4	ZrO_2_ = 30
	La_2_O_3_ = 7.7	La_2_O_3_ = 10
	Al_2_O_3_ = 8.7	Al_2_O_3_ = 10
CZ50LA_1000_	CuO = 50.0	CuO = 50
	ZrO_2_ = 28.4	ZrO_2_ = 30
	La_2_O_3_ = 8.0	La_2_O_3_ = 10
	Al_2_O_3_ = 9.5	Al_2_O_3_ = 10
Commercial catalyst HiFUEL W220 (HF220)		CuO = 52.5
		ZnO = 30.2
		Al_2_O_3_ = 17.0 Others = 0.3

**Table 5:** Reactivity results in comparison with equilibrium.

Parameter	HF220	CZ30LA_750_	CZ30LA_1000_	Equilibrium
H_2_O/CH_3_OH (1.0–3.0)	1.5	1.5	1.5	1.5
W/F_A0_ (kg s mol^−^^1^) (40–120)	100	100	100	-
Temperature (°C) (180–310)	246	246	246	246
CH_3_OH conversion (%)	97.0	89.1	87.7	99.9
CO selectivity (%)	3.7	1.7	1.4	4.9
H_2_ selectivity (%)	78.1	78.3	78.4	74.7
CO_2_ selectivity (%)	96.3	98.3	98.7	95.0

## References

[1] Claudio-Piedras A Ramírez-Zamora RM Alcántar-Vázquez BC Gutiérrez-Martínez A Mondragón-Galicia G One dimensional Pt/CeO_2_-NR catalysts for hydrogen production by steam reforming of methanol: Effect of Pt precursor Catalysis Today 2021 360 55 62 10.1016/j.cattod.2019.08.013

[2] Doğu T Varişli D Alcohols as alternatives to petroleum for environmentally clean fuels and petrochemicals Turkish Journal of Chemistry 2007 31 5 551 567

[3] Andersson J Grönkvist S Large-scale storage of hydrogen International Journal of Hydrogen Energy 2019 44 11901 11919 10.1016/j.ijhydene.2019.03.063

[4] Silva H Mateos-Pedrero C Ribeirinha P Boaventura M Mendes A Low-temperature methanol steam reforming kinetics over a novel CuZrDyAl catalyst Reaction Kinetics, Mechanisms, and Catalysis 2015 115 321 339 10.1007/s11144-015-0846-z

[5] Herdem MS Sinaki MY Farhad S Hamdullahpur F An overview of the methanol reforming process: Comparison of fuels, catalysts, reformers, and systems International Journal of Energy Research 2019 43 5076 5105 10.1002/er.4440

[6] Lima Da Silva A Müller IL Hydrogen production by sorption enhanced steam reforming of oxygenated hydrocarbons (ethanol, glycerol, n-butanol and methanol): Thermodynamic modelling International Journal of Hydrogen Energy 2011 36 2057 2075 10.1016/j.ijhydene.2010.11.051

[7] Zhang J Xiang Y Lu S Jiang SP High Temperature Polymer Electrolyte Membrane Fuel Cells for Integrated Fuel Cell – Methanol Reformer Power Systems: A Critical Review Advanced Sustainable Systems 2018 2 1 19 10.1002/adsu.201700184

[8] Khzouz M Wood J Pollet B Bujalski W Characterization and activity test of commercial Ni/Al_2_O_3_, Cu/ZnO/Al_2_O_3_ and prepared Ni-Cu/Al_2_O_3_ catalysts for hydrogen production from methane and methanol fuels International Journal of Hydrogen Energy 2013 38 1664 1675 10.1016/j.ijhydene.2012.07.026

[9] Ilsen Z Catalytic Processes for Clean Hydrogen Production Turkish Journal of Chemistry 2007 31 5 531 550

[10] Chein R Chen YC Chung JN Numerical study of methanol-steam reforming and methanol-air catalytic combustion in annulus reactors for hydrogen production Applied Energy 2013 102 1022 1034 10.1016/j.apenergy.2012.06.010

[11] Ouzounidou M Ipsakis D Voutetakis S Papadopoulou S Seferlis P A combined methanol autothermal steam reforming and PEM fuel cell pilot plant unit: Experimental and simulation studies Energy 2009 34 1733 1743 10.1016/j.energy.2009.06.031

[12] Pojanavaraphan C Luengnaruemitchai A Gulari E Effect of support composition and metal loading on Au catalyst activity in steam reforming of methanol International Journal of Hydrogen Energy 2012 37 14072 14084 10.1016/j.ijhydene.2012.06.107

[13] Suh JS Lee MT Greif R Grigoropoulos CP Transport phenomena in a steam-methanol reforming microreactor with internal heating International Journal of Hydrogen Energy 2009 34 314 322 10.1016/j.ijhydene.2008.09.049

[14] Lei Y Luo Y Li X Lu J Mei Z The role of samarium on Cu/Al_2_O_3_ catalyst in the methanol steam reforming for hydrogen production Catalysis Today 2018 307 162 168 10.1016/j.cattod.2017.05.072

[15] Men Y Kolb G Zapf R O’Connell M Ziogas A Methanol steam reforming over bimetallic Pd-In/Al_2_O_3_ catalysts in a microstructured reactor Applied Catalysis A: General 2010 380 15 20 10.1016/j.apcata.2010.03.004

[16] Sá S Silva H Brandão L Sousa JM Mendes A Catalysts for methanol steam reforming-A review Applied Catalysis B: Environmental 2010 99 43 57 10.1016/j.apcatb.2010.06.015

[17] Sanches SG Flores JH De Avillez RR Pais Da Silva MI Influence of preparation methods and Zr and y promoters on Cu/ZnO catalysts used for methanol steam reforming International Journal of Hydrogen Energy 2012 37 6572 6579 10.1016/j.ijhydene.2012.01.033

[18] Ahmadi F Haghighi M Ajamein H Sonochemically coprecipitation synthesis of CuO/ZnO/ZrO_2_/Al_2_O_3_ nanocatalyst for fuel cell grade hydrogen production via steam methanol reforming Journal of Molecular Catalysis A: Chemical 2016 421 196 208 10.1016/j.molcata.2016.05.027

[19] Byeon JH Kim YW Ultrasound-assisted copper deposition on a polymer membrane and application for methanol steam reforming Ultrasonics Sonochemistry 2013 20 472 477 10.1016/j.ultsonch.2012.06.002 22771243

[20] Fasanya OO Al-Hajri R Ahmed OU Myint MTZ Atta AY Copper zinc oxide nanocatalysts grown on cordierite substrate for hydrogen production using methanol steam reforming International Journal of Hydrogen Energy 2019 44 22936 22946 10.1016/j.ijhydene.2019.06.185

[21] Matsumura Y Durable Cu composite catalyst for hydrogen production by high temperature methanol steam reforming Journal of Power Sources 2014 272 961 969 10.1016/j.jpowsour.2014.09.047

[22] Pojanavaraphan C Luengnaruemitchai A Gulari E Effect of steam content and O_2_ pretreatment on the catalytic activities of Au/CeO_2_-Fe_2_O_3_ catalysts for steam reforming of methanol Journal of Industrial and Engineering Chemistry 2014 20 961 971 10.1016/j.jiec.2013.06.029

[23] Frank B Jentoft F Soerijanto H Krohnert J Schlogl R Steam reforming of methanol over copper-containing catalysts: Influence of support material on microkinetics Journal of Catalysis 2007 246 177 192 10.1016/j.jcat.2006.11.031

[24] Matsumura Y Ishibe H Effect of zirconium oxide added to Cu/ZnO catalyst for steam reforming of methanol to hydrogen Journal of Molecular Catalysis A: Chemical 2011 345 44 53 10.1016/j.molcata.2011.05.017

[25] Alejo L Lago R Peña MA Fierro JLG Partial oxidation of methanol to produce hydrogen over Cu-Zn-based catalysts Applied Catalysis A: General 1997 162 281 297 10.1016/S0926-860X(97)00112-9

[26] Behrens M Armbrüster M Methanol Steam Reforming Guczi L Erdóhelyi A Catalysis for Alternative Energy Generation New York, USA Springer 2012 175 235

[27] Stekrova M Rinta-Paavola A Karinen R Hydrogen production via aqueous-phase reforming of methanol over nickel modified Ce, Zr and La oxide supports Catalysis Today 2018 304 143 152 10.1016/j.cattod.2017.08.030

[28] Khani Y Tahay P Bahadoran F Safari N Soltanali S Synergic effect of heat and light on the catalytic reforming of methanol over Cu/x-TiO_2_(x=La, Zn, Sm, Ce) nanocatalysts Applied Catalysis A: General 2020 594 117456 10.1016/j.apcata.2020.117456

[29] Papavasiliou J Avgouropoulos G Ioannides T Effect of dopants on the performance of CuO-CeO_2_ catalysts in methanol steam reforming Applied Catalysis B: Environmental 2007 69 226 234 10.1016/j.apcatb.2006.07.007

[30] Lu J Li X He S Han C Wan G Hydrogen production via methanol steam reforming over Ni-based catalysts: Influences of Lanthanum (La) addition and supports International Journal of Hydrogen Energy 2017 42 3647 3657 10.1016/j.ijhydene.2016.08.165

[31] Aspen Technology Inc Website https://www.aspentech.com/ accessed 22 February 2022

[32] Gražulis S Chateigner D Downs RT Yokochi AFT Quirós M Crystallography Open Database – an open-access collection of crystal structures Journal of Applied Crystallograpghy 2009 42 726 729 10.1107/S0021889809016690 PMC3253730 22477773

[33] Özcan O Akın AN Thermodynamic analysis of methanol steam reforming to produce hydrogen for HT-PEMFC: An optimization study International Journal of Hydrogen Energy 2019 44 14117 14126 10.1016/j.ijhydene.2018.12.211

[34] Özcan MD Özcan O Akın AN Thermodynamic modelling and optimization of oxy-reforming and oxy-steam reforming of biogas by RSM Environmental Technology 2020 41 14 28 10.1080/09593330.2019.1639828 31264942

[35] Ribeirinha P Mateos-Pedrero C Boaventura M Sousa J Mendes A CuO/ZnO/Ga_2_O_3_ catalyst for low temperature MSR reaction: Synthesis, characterization and kinetic model Applied Catalysis B: Environmental 2018 221 371 379 10.1016/j.apcatb.2017.09.040

[36] Varmazyari M Khani Y Bahadoran F Shariatinia Z Soltanali S Hydrogen production employing Cu(BDC) metal–organic framework support in methanol steam reforming process within monolithic micro-reactors International Journal of Hydrogen Energy 2021 46 565 580 10.1016/j.ijhydene.2020.09.245

